# Development and validation of a machine learning model to evaluate survival in patients with newly diagnosed breast cancer with liver metastasis

**DOI:** 10.3389/fonc.2026.1777850

**Published:** 2026-06-16

**Authors:** Yao Wang, Yu Yue, Xu-Chen Cao

**Affiliations:** 1 The First Department of Breast Cancer, Tianjin Medical University Cancer Institute and Hospital, National Clinical Research Center for Cancer, Tianjin, China; 2 Key Laboratory of Cancer Prevention and Therapy, Tianjin, China; 3 Tianjin’s Clinical Research Center for Cancer, Tianjin, China; 4 Key Laboratory of Breast Cancer Prevention and Therapy, Tianjin Medical University, Ministry of Education, Tianjin, China

**Keywords:** artificial intelligence, breast cancer, liver metastasis, nomogram, prognostic model

## Abstract

**Background:**

Breast cancer liver metastasis (BCLM) is associated with substantial prognostic heterogeneity and poor survival outcomes. Existing staging systems and prognostic models have a limited capacity for individualized survival prediction for patients with newly diagnosed BCLM, highlighting the need for more accurate and clinically applicable predictive tools. This study aimed to develop a prognostic nomogram for patients with newly diagnosed breast cancer and liver metastases using Surveillance, Epidemiology, and End Results (SEER) database data (2010–2021).

**Methods:**

Ten stable prognostic variables—age; tumor size; brain, bone, and lung metastases; histological grade; chemotherapy; estrogen receptor (ER) status; progesterone receptor (PR) status; and human epidermal growth factor receptor-2 (HER2) status—were selected using four machine learning algorithms (Least Absolute Shrinkage And Selection Operator, Extreme Gradient Boosting, Decision Tree, and Random Forest). The SEER cohort was randomly divided into training (70%) and internal validation (30%) cohorts, with an independent external cohort for validation. These variables were incorporated into a multivariate Cox proportional hazards regression model to construct a nomogram. Model performance was evaluated using the concordance index (C-index), calibration curves, decision curve analysis (DCA), and area under the curve (AUC).

**Results:**

The C-indices for the training, internal validation, and external validation cohorts were 0.760, 0.740, and 0.787, respectively. The 1-, 3-, and 5-year AUCs were 0.777, 0.757, and 0.764 (training); 0.755, 0.769, and 0.754 (internal validation); and 0.727, 0.752, and 0.801 (external validation), respectively. The calibration curves indicated good agreement and DCA confirmed the clinical utility of the model.

**Conclusion:**

The proposed nomogram demonstrated robust predictive performance for patients with breast cancer and liver metastases and may provide valuable prognostic information, pending further validation.

## 1 Introduction

An estimated 2,308,000 new breast cancer cases have been documented globally in 2022, accounting for 11.6% of all newly diagnosed cancers worldwide. Breast cancer has the highest incidence among malignancies in women (1). The 5-year cumulative risks of progression from early-stage to metastatic breast cancer were 3.7% for stage I, 13.3% for stage II, and 30.9% for stage III disease. Additionally, 20–30% of patients with early-stage breast cancer may eventually develop metastatic disease (2). A large Surveillance, Epidemiology, and End Results (SEER)-based study reported that approximately 3–10% of patients with breast cancer present with stage IV disease at initial diagnosis (3). Furthermore, approximately 5–10% of patients present with *de novo* stage IV breast cancer (4). Metastatic cancer is associated with poor survival outcomes and prognosis.

Breast cancer commonly metastasizes to the brain, lungs, liver, and bones through hematogenous spread. The liver is the third most common metastatic site in breast cancer, with solid tumors frequently spreading to this organ (5). Patients with breast cancer with liver metastases (BCLM) have a median survival of approximately 20 months, with prognosis varying substantially across molecular subtypes. Notably, the triple-negative subtype is associated with the poorest prognosis (6). The unique clinical challenges and poor outcomes associated with BCLM highlight the need for tailored prognostic models to improve risk stratification and treatment decision-making in this high-risk population.

Prognostic assessment in advanced breast cancer currently relies primarily on the TNM staging system, which evaluates tumor size, lymph node involvement, and distant metastasis. Although clinically useful, the TNM system categorizes liver metastasis only as part of M1 disease and does not account for the substantial prognostic heterogeneity among patients with BCLM (7, 8). Moreover, the TNM system does not incorporate several important prognostic determinants, including demographic characteristics, molecular subtype, tumor biology, extrahepatic metastatic burden, and treatment-related factors, all of which are increasingly recognized as essential for individualized survival prediction. Although several breast cancer-specific nomograms have been developed for prognostic prediction, most were designed for general breast cancer populations rather than specifically for patients with BCLM. More recently, SEER-based nomograms focused on BCLM have been reported (9), reflecting growing interest in individualized prognostic prediction for this subgroup. However, existing models have largely relied on conventional statistical approaches, and independent external validation remains limited, potentially restricting their generalizability and clinical applicability.

This limitation represents an important unmet clinical and research need. Patients with BCLM exhibit substantial survival heterogeneity according to molecular subtype, extrahepatic metastatic burden, tumor biology, and treatment response, making prognosis difficult to estimate using conventional staging systems or general breast cancer nomograms (6, 7, 9, 10). In clinical practice, the absence of a BCLM-specific predictive model may limit individualized treatment planning, follow-up strategies, and patient counseling. Furthermore, many existing prognostic models rely on conventional regression-based variable selection methods and lack validation in independent external cohorts, which may further limit their generalizability. To address these gaps, this study aimed to develop a prognostic model specifically for newly diagnosed patients with BCLM by integrating machine learning-based feature selection with multivariate Cox proportional hazards regression. Using contemporary SEER data from 2010 to 2021 and an independent external validation cohort, this study aimed to construct a clinically applicable model that integrates demographic, clinicopathological, metastatic, treatment-related, and molecular factors to support individualized survival prediction beyond conventional staging systems and existing general breast cancer nomograms.

## 2 Materials and methods

### 2.1 Data collection

**Figure 1:**
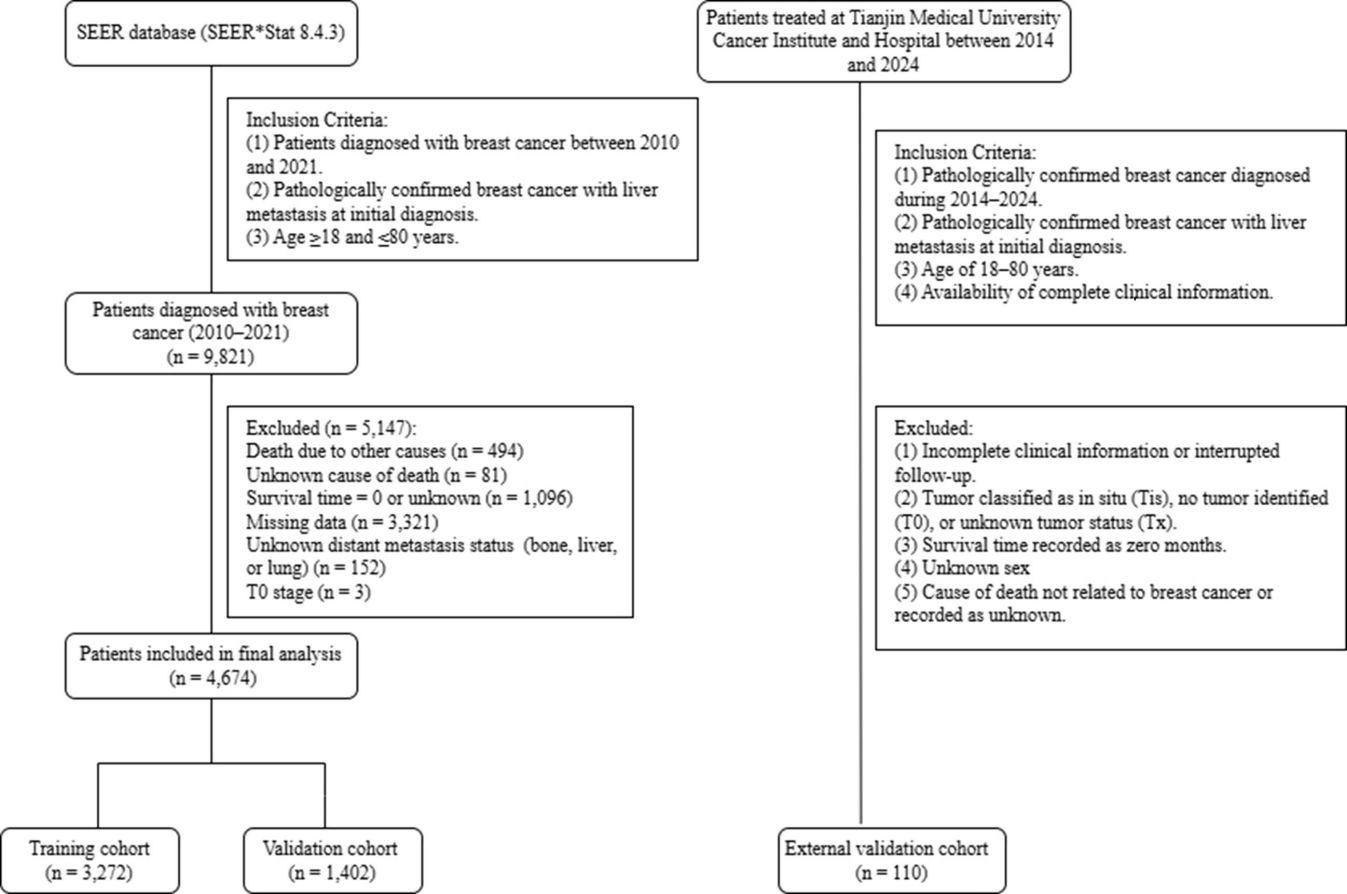
Flowchart of patient selection. Selection process of patients with newly diagnosed BCLM from the SEER database between 2010 and 2021 and the inclusion of an independent external validation cohort from Tianjin Medical University Cancer Institute and Hospital between 2014 and 2024. After applying the predefined inclusion and exclusion criteria, 4,674 eligible patients are included in the final SEER cohort and randomly divided into the training (n = 3,272) and internal validation (n = 1,402) cohorts at a 7:3 ratio, respectively. The independent external validation cohort includes 110 patients and was used exclusively for external validation of the prognostic model. BCLM, breast cancer liver metastasis; SEER, Surveillance, Epidemiology, and End Results.

This retrospective study included patients with BCLM from the US SEER database (2010–2021). The initial SEER cohort comprised 9,821 patients. Based on the inclusion and exclusion criteria, 5,147 patients were excluded from the study. The final SEER cohort included 4,674 patients randomly assigned to a training cohort (n = 3,272; 70%) or an internal validation cohort (n = 1,402; 30%) (**Figure 1**). An independent cohort of 110 patients from Tianjin Medical University Cancer Institute and Hospital (2014–2024) served as the external validation cohort. This cohort was geographically and institutionally distinct from the SEER cohort and was not used for model training or internal validation. Patients with isolated liver metastases and concomitant extrahepatic metastases were included in the study.

### 2.2 Inclusion and exclusion criteria

We included patients with the following: (1) Pathologically confirmed breast cancer diagnosed between 2010 and 2021; (2) pathologically confirmed breast cancer with documented liver metastases at the initial diagnosis; (3) age of 18–80 years; and (4) availability of complete clinical information. We excluded patients with (1) incomplete clinical information or interrupted follow-up; (2) Tumor classified as *in situ* (Tis), no identified tumor (T0), or unknown tumor status (Tx); (3) survival time recorded as 0 months; (4) unknown sex; and (5) cause of death unrelated to breast cancer or unknown.

Because the SEER database records metastatic status only at the time of initial diagnosis and does not provide information on disease recurrence, we included patients with breast cancer who presented with liver metastasis at diagnosis, operationally defined as *de novo* BCLM. Patients who developed liver metastasis after the initial diagnosis of breast cancer could not be reliably identified using the SEER; therefore, they were not included in the study population. Patients with isolated liver metastases and those with concomitant extrahepatic metastases were eligible. This design allowed us to focus on newly identified BCLM and develop a prognostic model based on key clinical and molecular characteristics associated with liver metastatic involvement. Overall survival (OS) was defined as the interval from the date of diagnosis to death from any cause, with surviving patients censored at the last follow-up. Estrogen receptor (ER) and progesterone receptor (PR) positivity status were defined as nuclear staining in ≥1% of invasive tumor cells by immunohistochemistry (IHC). Human epidermal growth factor receptor 2 (HER2) status positivity was defined as an IHC score of 3+ or an IHC score of 2+, with gene amplification confirmed by *in situ* hybridization (ISH); all other results were considered negative.

### 2.3 Statistical analyses

Continuous variables are summarized as mean ± standard deviation or median with interquartile range (IQR), as appropriate. Categorical variables are presented as frequencies and percentages. Comparisons among the SEER training cohort, SEER internal validation cohort, and independent external validation cohort were performed using one-way analysis of variance or the Kruskal–Wallis test for continuous variables and the chi-square test or Fisher’s exact test for categorical variables, as appropriate. When an overall difference was observed, pairwise comparisons were conducted using Bonferroni correction to adjust for multiple testing.

The SEER cohort was randomly divided into training and internal validation cohorts at a ratio of 7:3. The institutional cohort from the Tianjin Medical University Cancer Institute and Hospital served as an independent external validation cohort and was not involved in model training or internal validation.

The analytical workflow comprised three sequential steps: feature selection, model construction, and model validation. Four machine learning algorithms were applied to the training cohort for feature selection: Least Absolute Shrinkage and Selection Operator (LASSO), Extreme Gradient Boosting (XGBoost), Decision Tree (DT), and Random Forest (RF). These algorithms were used solely for variable selection and not for final model construction. Only the variables consistently identified by all four algorithms were retained as stable candidate predictors. These variables were then entered into a multivariate Cox proportional hazards regression model to determine the independent prognostic factors and construct the final prognostic nomogram. Cox regression was selected because it is suitable for time-to-event outcomes with censored observations, provides interpretable hazard ratios (HRs) with 95% confidence intervals (CIs), and can be readily translated into a clinically applicable nomogram for individualized survival prediction. The predictive performance of the nomogram was evaluated using concordance index (C-index), time-dependent receiver operating characteristic (ROC) curves, calibration curves, and decision curve analysis (DCA).

### 2.4 Feature selection

To identify robust prognostic variables and reduce potential selection bias, four machine learning algorithms—LASSO, DT, RF, and XGBoost—were applied independently to the SEER training cohort. Each algorithm evaluated candidate variables according to specific criteria. In the LASSO model, variables with nonzero regression coefficients were retained. For the tree-based models (DT, RF, and XGBoost), the variables were ranked based on their importance scores.

**Figure 2:**
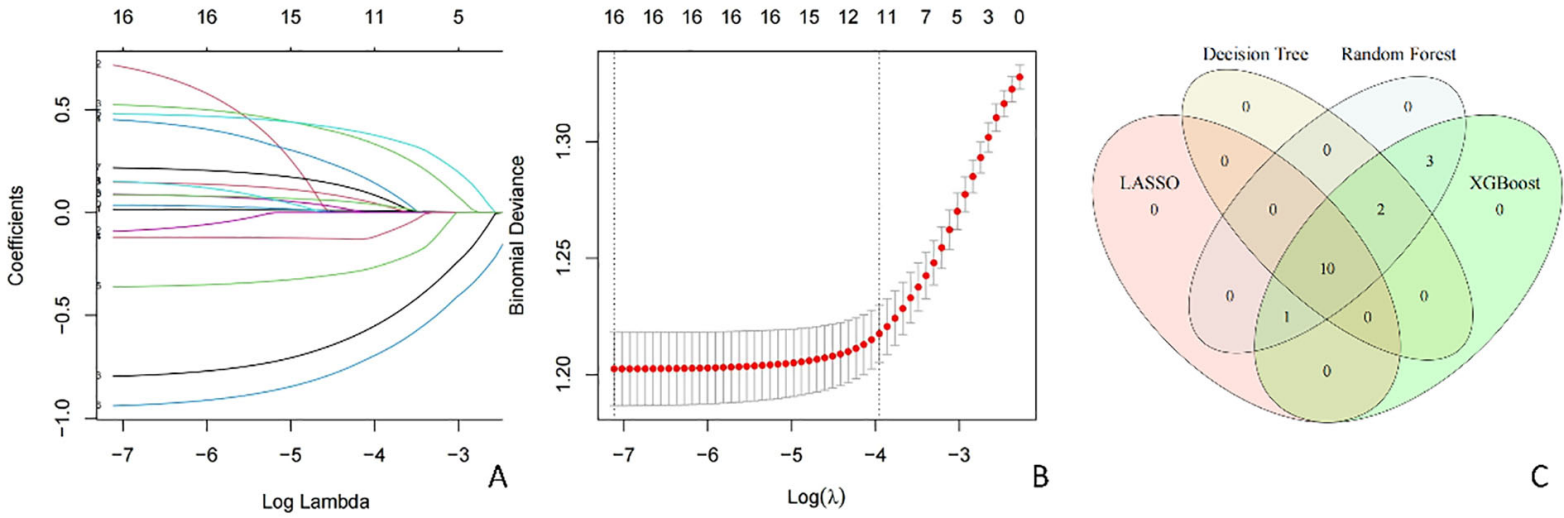
Analytical workflow for feature selection, nomogram construction, and validation. **(A)** LASSO coefficient profiles of candidate variables. **(B)** Ten-fold cross-validation for tuning parameter selection in the LASSO model. **(C)** Venn diagram showing the overlap of variables selected by LASSO, XGBoost, Decision Tree (DT), and Random Forest (RF) algorithms. The SEER cohort was divided into training and internal validation cohorts, and an independent institutional cohort was used for external validation. Four machine learning algorithms (LASSO, XGBoost, DT, and RF) were applied only for feature selection in the training cohort. Variables consistently selected by all four algorithms were entered into a multivariable Cox proportional hazards regression model to construct the final nomogram for overall survival (OS) prediction. Model performance was evaluated using the C-index, time-dependent ROC curves, calibration curves, and decision curve analysis (DCA). DCA, decision curve analysis; DT, Decision Tree; LASSO, Least Absolute Shrinkage and Selection Operator; OS, overall survival; RF, Random Forest; ROC, receiver operating characteristic; SEER, Surveillance, Epidemiology, and End Results; XGBoost, Extreme Gradient Boosting.

The overlap between the features selected by the four algorithms was visualized using a Venn diagram (**Figure 2**). To ensure stability and consistency, only the variables identified by all four algorithms were retained as stable candidate predictors. Variables selected using only one, two, or three algorithms were excluded from further analysis. Based on this strict intersection criterion, ten variables were selected.

These 10 stable candidate variables were subsequently incorporated into a multivariate Cox proportional hazards regression model to identify independent prognostic factors and construct the final nomogram. This approach ensures that the final model is built on features that are consistently recognized across multiple machine learning methods, thereby enhancing robustness and reducing algorithm-specific bias.

## 3 Results

### 3.1 Patient baseline characteristics

A total of 4,784 patients with BCLM were included, comprising 3,272 in the SEER training cohort, 1,402 in internal validation cohort, and 110 in external validation cohort from the Tianjin Medical University Cancer Institute and Hospital (2014–2024), which were geographically and institutionally distinct from the SEER data. The median age was similar across cohorts (training/internal: 56.0 [47–65] years; external: 52.0 [44–60] years), and over 99% of the patients were female. Bone, brain, and lung metastases were observed in 57–58%, 6–9%, and 24–31% of patients, respectively, with bone metastases significantly less frequent in the external cohort (P < 0.001). Breast cancer subtypes and histological grades showed modest variability, with HR−/HER2+ tumors and lower rates of Grade III+ tumors being more prevalent in the external cohort. The T- and N-stage distributions were broadly comparable, whereas treatment patterns, including primary surgery, radiotherapy, and chemotherapy, were largely consistent, although surgery and radiotherapy rates were lower in the external cohort. Median OS was 20.0 months in SEER cohorts and 17.5 months in the external cohort, with survival status distributions reflecting similar trends (**Table 1**). Despite inter-cohort differences and partial temporal overlap with the SEER data, the geographic independence and consistent distribution of key prognostic variables of the external cohort support the generalizability of the developed nomogram across independent populations. Building on these cohort characteristics, 10 prognostic variables were used to construct a Cox regression-based nomogram for individualized survival prediction across all cohorts.

**Table 1:** Clinicopathologic and baseline characteristics of patients with BCLM.

Variable	ALL(N = 4784)	Internal validation cohort(N = 1402)	Training cohort(N = 3272)	External validation cohort (N = 110)	P value (Overall)
	N=4784	N=1402	N=3272	N=110	
Age (years)	56.0 [47.0-65.0]	56.0 [47.0-65.0]	56.0 [47.0-65.0]	52.0 [44.0-60.0]	0.005
Gender, n (%)					1.000
Female	4758 (99.5)	1394 (99.4)	3254 (99.4)	110 (100.0)	
Male	26 (0.5)	8 (0.6)	18 (0.6)	0 (0.0)	
Bone metastases, n (%)					<0.001
No	2014 (42.1)	594 (42.4)	1353 (41.4)	67 (60.9)	
Yes	2770 (57.9)	808 (57.6)	1919 (58.6)	43 (39.1)	
Brain metastases, n (%)					0.671
No	4378 (91.5)	1279 (91.2)	2996 (91.6)	103 (93.6)	
Yes	406 (8.5)	123 (8.8)	276 (8.4)	7 (6.4)	
Lung metastases, n (%)					0.191
No	3306 (69.1)	977 (69.7)	2245 (68.6)	84 (76.4)	
Yes	1478 (30.9)	425 (30.3)	1027 (31.4)	26 (23.6)	
Breast subtype, n (%)					0.001
HR+/HER2-	2080 (43.5)	603 (43.0)	1438 (43.9)	39 (35.5)	
HR+/HER2+	1174 (24.5)	340 (24.3)	808 (24.7)	26 (23.6)	
HR-/HER2+	805 (16.8)	242 (17.3)	527 (16.1)	36 (32.7)	
HR-/HER2-	725 (15.2)	217 (15.5)	499 (15.3)	9 (8.2)	
Grade, n (%)					<0.001
Grade I	237 (5.0)	67 (4.8)	132 (4.0)	38 (34.5)	
Grade II	1842 (38.5)	538 (38.4)	1260 (38.5)	44 (40.0)	
Grade III+	2705 (56.5)	797 (56.8)	1880 (57.5)	28 (25.5)	
Histology, n (%)					0.007
Infiltrating duct carcinoma	4032 (84.3)	1178 (84.0)	2755 (84.2)	99 (90.0)	
Lobular carcinoma	297 (6.2)	89 (6.4)	197 (6.0)	11 (10.0)	
Other	455 (9.5)	135 (9.6)	320 (9.8)	0 (0.0)	
T Stage, n (%)					0.049
T1	590 (12.3)	168 (12.0)	418 (12.8)	4 (3.6)	
T2	1683 (35.2)	504 (35.9)	1129 (34.5)	50 (45.5)	
T3	926 (19.4)	262 (18.7)	640 (19.6)	24 (21.8)	
T4	1585 (33.1)	468 (33.4)	1085 (33.2)	32 (29.1)	
N Stage, n (%)					<0.001
N0	914 (19.1)	250 (17.8)	664 (20.3)	0 (0.0)	
N1	2519 (52.7)	787 (56.1)	1695 (51.8)	37 (33.6)	
N2	580 (12.1)	150 (10.7)	383 (11.7)	47 (42.7)	
N3	771 (16.1)	215 (15.3)	530 (16.2)	26 (23.6)	
Primary surgery, n (%)					0.001
No	3525 (73.7)	1049 (74.8)	2379 (72.7)	97 (88.2)	
Yes	1259 (26.3)	353 (25.2)	893 (27.3)	13 (11.8)	
Radiation, n (%)					<0.001
No	3385 (70.8)	1005 (71.7)	2276 (69.6)	104 (94.5)	
Yes	1399 (29.2)	397 (28.3)	996 (30.4)	6 (5.5)	
Chemotherapy, n (%)					0.010
No	888 (18.6)	250 (17.8)	629 (19.2)	9 (8.2)	
Yes	3896 (81.4)	1152 (82.2)	2643 (80.8)	101 (91.8)	
ER status, n (%)					0.149
Negative	1643 (34.3)	494 (35.2)	1103 (33.7)	46 (41.8)	
Positive	3141 (65.7)	908 (64.8)	2169 (66.3)	64 (58.2)	
PR status, n (%)					0.050
Negative	2371 (49.6)	684 (48.8)	1620 (49.5)	67 (60.9)	
Positive	2413 (50.4)	718 (51.2)	1652 (50.5)	43 (39.1)	
HER2 status, n (%)					0.003
Negative	2804 (58.6)	820 (58.5)	1937 (59.2)	47 (42.7)	
Positive	1980 (41.4)	582 (41.5)	1335 (40.8)	63 (57.3)	
Survival months	20.0 [8.0;41.0]	20.0 [7.0;43.8]	20.0 [8.0;41.0]	17.5 [9.0;24.0]	0.086
Survival status, n (%)					<0.001
Alive	1853 (38.7)	528 (37.7)	1244 (38.0)	81 (73.6)	
Dead	2931 (61.3)	874 (62.3)	2028 (62.0)	29 (26.4)	

ER, estrogen receptor; PR, progesterone receptor; HER2, human epidermal growth factor receptor 2; Grade III+, grade III or IV. Continuous variables are presented as median [interquartile range], and categorical variables are presented as n (%). P values indicate comparisons among the training, internal validation, and external validation cohorts. A two-sided P < 0.05 was considered statistically significant.

### 3.2 Construction and validation of the prognostic nomogram model

**Figure 3:**
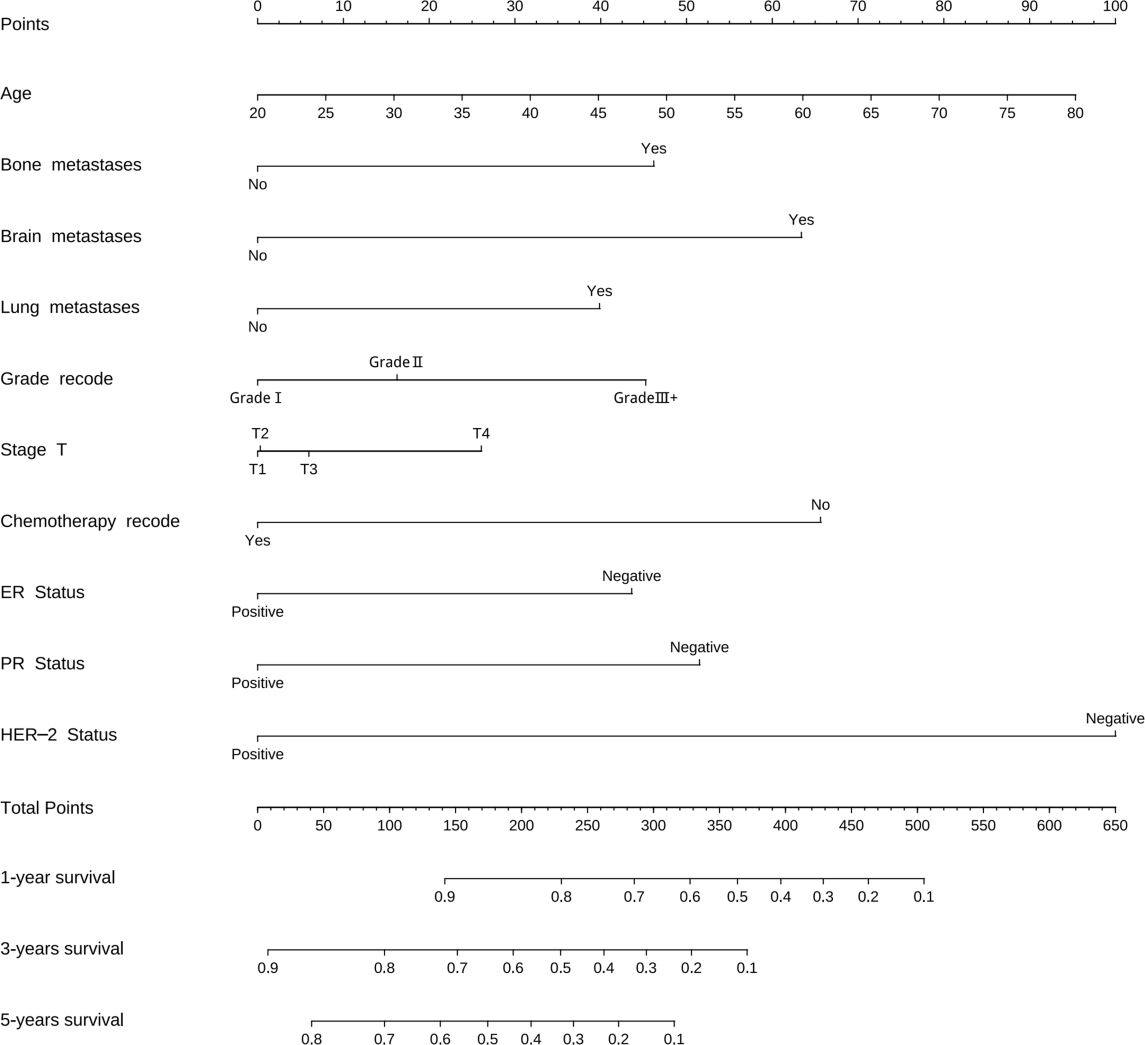
Nomogram construction. The nomogram, constructed from variables selected by four ML algorithms and incorporated into a multivariable Cox model, is evaluated in the training, internal validation, and external validation cohorts. Predictive performance was assessed using the C-index, time-dependent ROC curves, calibration curves, and DCA. Patients are stratified into low-, intermediate-, and high-risk groups based on nomogram-derived scores for OS prediction. BCLM, breast cancer liver metastasis; C-index, concordance index; DCA, decision curve analysis; OS, overall survival.

A prognostic nomogram was constructed using ten independent variables—age; tumor size; bone, brain, and lung metastases; histological grade; chemotherapy; and ER, PR, and HER2 status—selected by four machine learning algorithms and confirmed through multivariate Cox regression (**Tables 2**, **3**, **Figure 3**). Older age, larger tumors, and the presence of metastases were associated with worse survival, whereas chemotherapy and a positive hormone receptor status were protective. HER2 positivity was associated with an increased risk. Each variable contributed to a total score, which estimated the 1-, 3-, and 5-year OS rates, with higher scores indicating a poorer prognosis.

**Table 2:** Feature selection results from four machine learning algorithms.

Clinical feature	RF Weights	DT Weights	XGBoost Weights	LASSO Coefficient
AGE	0.304	0.098	0.184	0.008
SEX	0.004	0.000	0.002	0.000
METS AT DX-BONE	0.045	0.040	0.085	0.326
METS AT DX-BRAIN	0.024	0.020	0.022	0.134
METS AT DX-LUNG	0.045	0.080	0.100	0.372
SUB TYPE	0.042	0.042	0.027	0.000
GRADE	0.064	0.020	0.045	0.078
HIST/BEHAV	0.046	0.000	0.024	0.041
TUMOR SIZE	0.099	0.011	0.042	0.038
LYMPH NODE INVASION	0.091	0.010	0.055	0.000
SURG PRIM SITE	0.043	0.000	0.012	0.000
RADIATION	0.043	0.000	0.006	0.000
CHEMOTHERAPY	0.043	0.101	0.119	-0.541
ER STATUS	0.022	0.018	0.025	-0.111
PR STATUS	0.036	0.030	0.064	-0.260
HER2 STATUS	0.050	0.044	0.188	-0.686

ER, estrogen receptor; PR, progesterone receptor; HER2, human epidermal growth factor receptor 2.

**Table 3:** Multivariable Cox proportional hazards regression analysis.

Clinical feature	β	HR (95% CI)	P value
AGE	0.013	1.014(1.010-1.017)	<0.001
Mets at DX-bone			
NO		Reference	
YES	0.392	1.480(1.346-1.628)	<0.001
Mets at DX-brain			
NO		Reference	
YES	0.538	1.713(1.479-1.983)	<0.001
Mets at DX-lung			
NO		Reference	
YES	0.339	1.403(1.276-1.543)	<0.001
Grade			
I		Reference	
II	0.138	1.148(0.912-1.445)	0.256
III+	0.384	1.469(1.166-1.85)	0.002
T stage			
T1		Reference	
T2	0.003	1.003(0.866-1.161)	0.974
T3	0.051	1.052(0.896-1.235)	0.549
T4	0.222	1.248(1.079-1.443)	0.005
Chemotherapy			
NO		Reference	
YES	-0.557	0.573(0.513-0.639)	<0.001
ER status			
NEGATIVE		Reference	
POSITIVE	-0.370	0.690(0.610-0.782)	<0.001
PR status			
NEGATIVE		Reference	
POSITIVE	-0.438	0.646(0.574-0.727)	<0.001
HER2 status			
NEGATIVE		Reference	
POSITIVE	-0.849	0.428(0.387-0.473)	<0.001

ER, estrogen receptor; PR, progesterone receptor; HER2, human epidermal growth factor receptor 2; Grade III+, grade III or IV; HR, hazard ratio; CI, confidence interval. A two-sided P < 0.05 was considered statistically significant.

**Figure 4:**
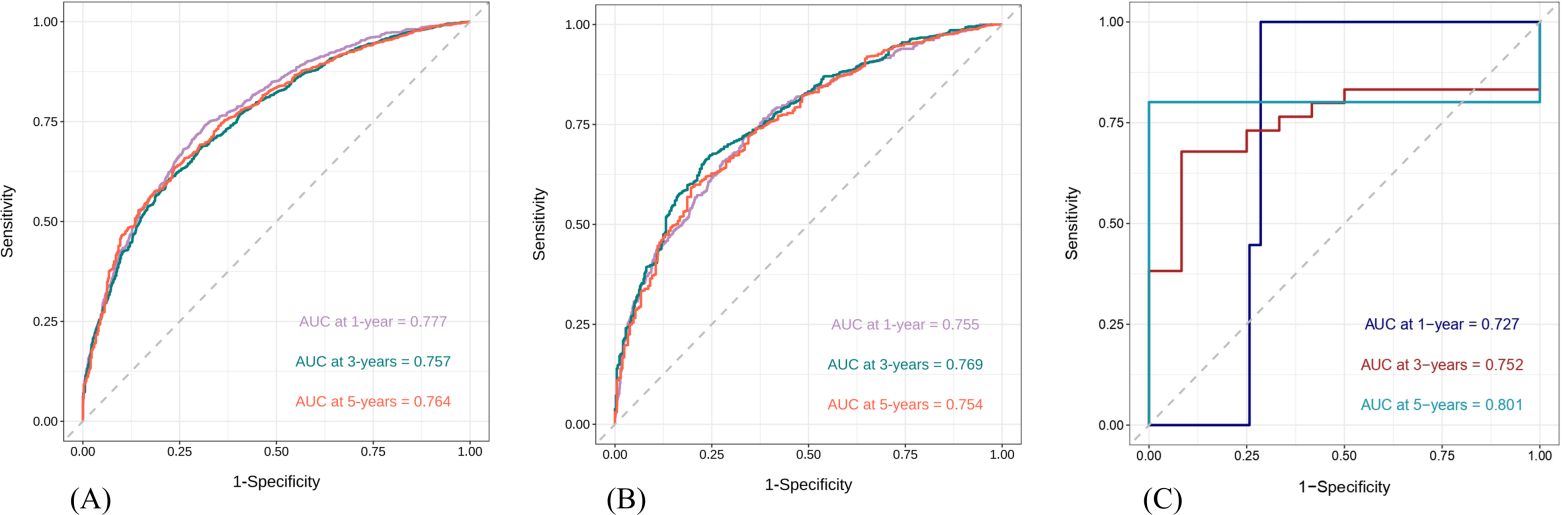
Time-dependent ROC curves were used to assess the discriminatory performance of the prognostic model for 1-, 3-, and 5-year OS in patients with BCLM. Panel **(A)** shows results for the training cohort, panel **(B)** for the internal validation cohort, and panel **(C)** for the external validation cohort. The AUC at each time point indicates the model’s ability to distinguish patients with different survival outcomes, demonstrating consistent predictive performance across all cohorts. AUC, The area under the ROC curve; BCLM, breast cancer liver metastasis; C-index, concordance index; DCA, decision curve analysis; OS, overall survival.

The model demonstrates robust discrimination across cohorts. C-index values were 0.760 (95% CI: 0.741–0.779) in the training cohort, 0.740 (95% CI: 0.718–0.762) in internal validation, and 0.787 (95% CI: 0.710–0.864) in the external validation cohort of 110 patients from Tianjin Medical University Cancer Institute and Hospital. Time-dependent ROC analyses yielded 1-, 3-, and 5-year AUCs above 0.70 in all cohorts (**Figure 4**), confirming stable predictive performance. Although the external cohort partially overlaps temporally with the SEER data, its geographic and institutional independence supports external validation, although minor temporal bias should be considered.

**Figure 5:**
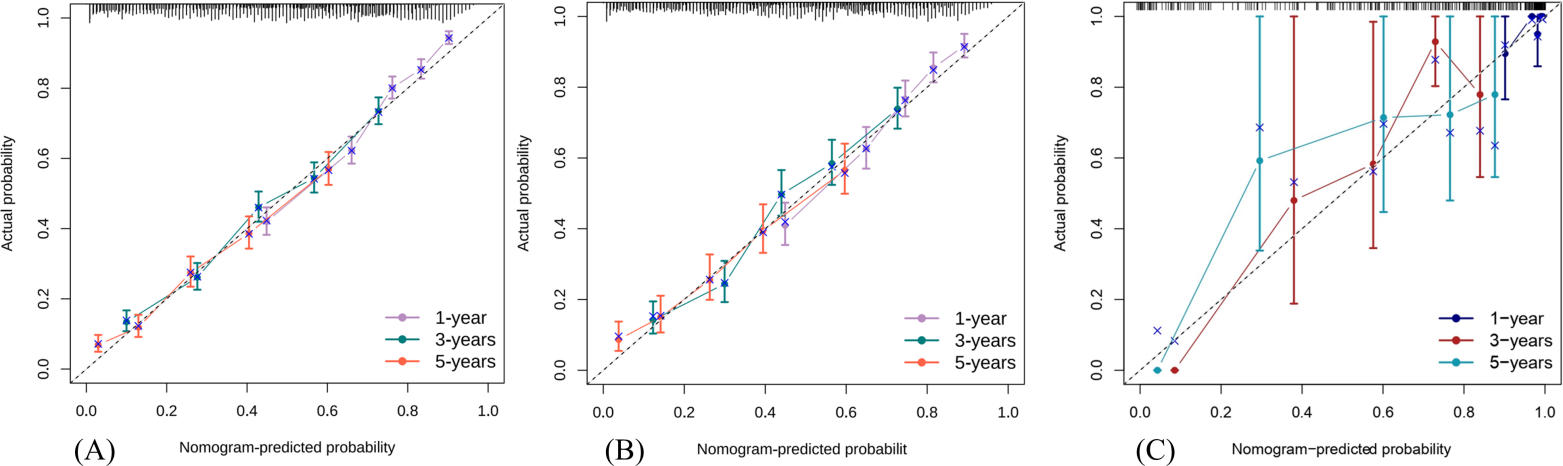
Calibration curves for the nomogram model, which predicts survival rates for BCLM patients at 1, 3, and 5 years. The x-axis represents the nomogram-predicted probability of OS, while the y-axis shows the actual OS calculated using the Kaplan–Meier method. Greater accuracy in predictions is reflected by points being closer to the 45-degree line. **(A)** Training cohort; **(B)** Internal validation cohort; **(C)** External validation cohort. BCLM, breast cancer liver metastasis; OS, overall survival.

**Figure 6:**
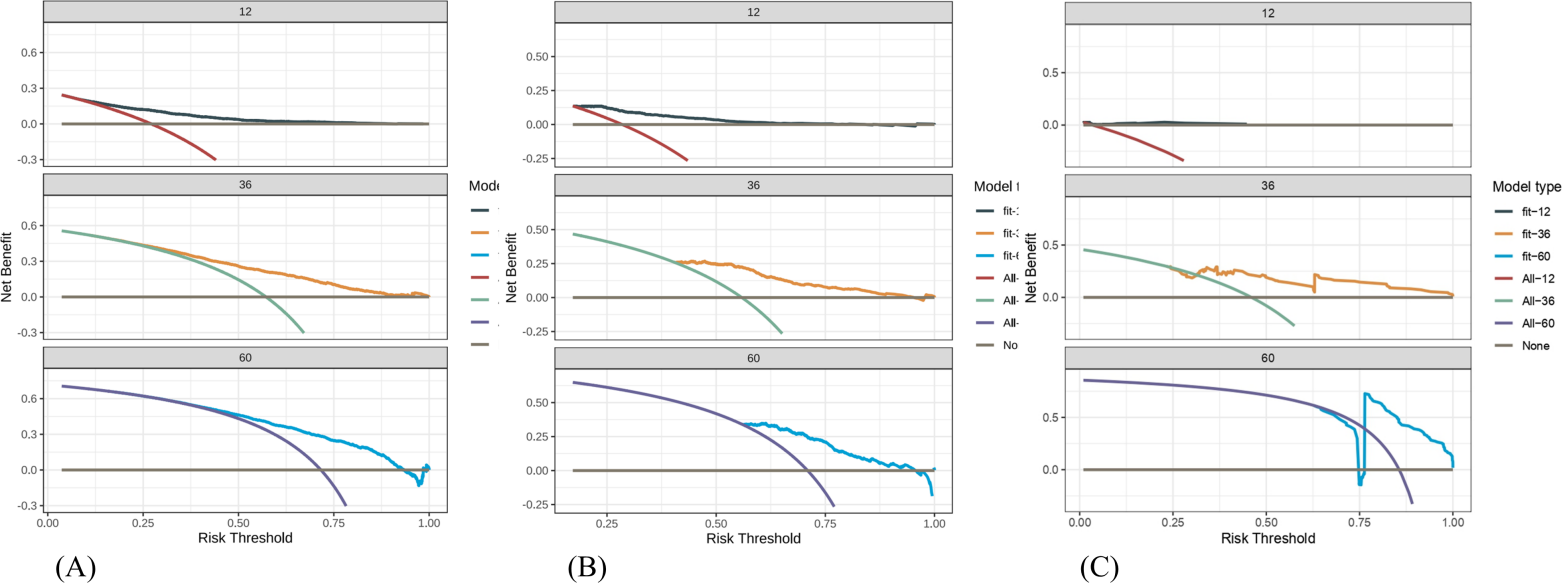
DCA of the prognostic nomogram for 1-, 3-, and 5-year OS in patients with newly diagnosed BCLM. The net benefit of the nomogram (colored lines) is compared with the “treat all” and “treat none” strategies (gray and black lines) across clinically relevant risk thresholds. Panels depict: **(A)** training cohort, **(B)** internal validation cohort, and **(C)** external validation cohort. The nomogram consistently demonstrates superior net benefit across all time points and cohorts, supporting its potential utility for individualized survival prediction and clinical decision-making in BCLM patients. BCLM, breast cancer liver metastasis; DCA, decision curve analysis; OS, overall survival.

Calibration curves indicated good agreement between predicted and observed OS across all cohorts (**Figure 5**). DCA demonstrated a net clinical benefit across a wide range of threshold probabilities (**Figure 6**), with minor limitations at 1 year in the external cohort, likely reflecting the smaller sample size.

**Figure 7:**
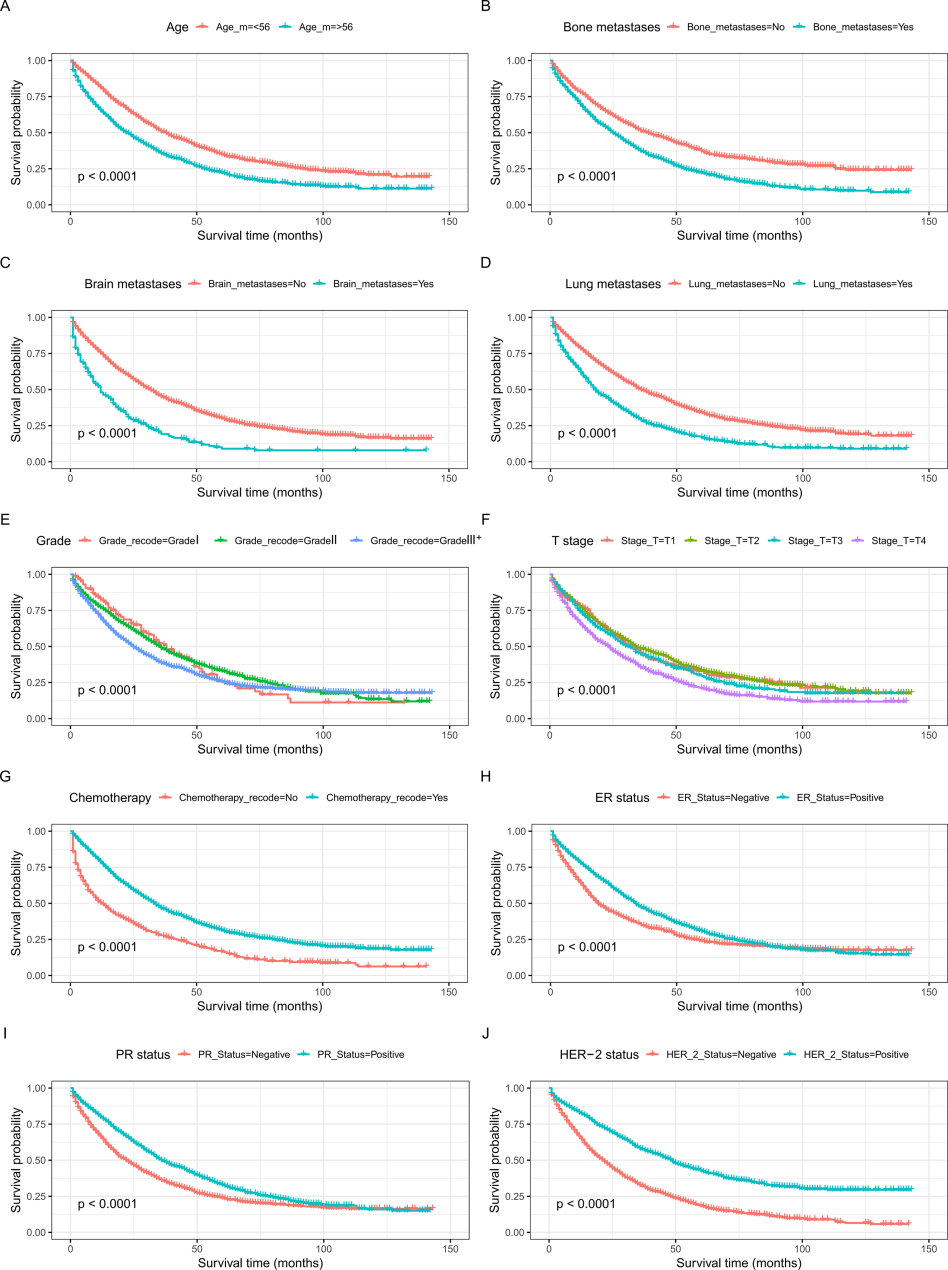
Kaplan–Meier curves for overall survival in BCLM patients stratified by prognostic factors. Panels show OS by **(A)** age, **(B)** bone metastases, **(C)** brain metastases, **(D)** lung metastases, **(E)** histological grade, **(F)** T stage, **(G)** chemotherapy, **(H)** ER status, **(I)** PR status, and **(J)** HER2 status. Subgroups are indicated in the legends; all differences were significant by log-rank test (p < 0.0001). Younger age, absence of organ metastases, lower grade and stage, receipt of chemotherapy, and positive receptor status were associated with improved survival. BCLM, breast cancer liver metastasis; HER2, human epidermal growth factor receptor 2; ER, estrogen receptor; OS, overall survival; PR, progesterone receptor.

Kaplan–Meier analyses further illustrated the impact of key prognostic factors (**Figure 7**). Younger patients (<56 years) had the following: significantly longer OS; bone, brain, and lung metastases; higher histological grade; advanced T stage; and HER2 positivity were associated with poorer survival, whereas ER/PR positivity conferred protection. Survival trends were consistent with the nomogram predictions across cohorts, demonstrating the model’s ability to stratify patients by risk. Notably, the nomogram improved discrimination compared to baseline risk stratification, supporting its potential clinical utility.

The nomogram showed reliable calibration, strong discrimination, and clinically meaningful net benefit overall. The integration of Cox regression with machine learning-based variable selection coupled with external validation confirmed its utility for individualized survival prediction and risk stratification in newly diagnosed BCLM patients.

## 4 Discussion

This study leveraged a large clinical dataset from the SEER database and applied multiple machine learning algorithms to identify 10 independent prognostic factors in patients with BCLM. A prognostic nomogram was constructed using the variables and was internally validated with further external validation in an independent cohort. Older age, larger tumors, multisite metastases, higher histological grade, lack of chemotherapy, and negative hormone receptor or HER2 status were associated with increased mortality, consistent with established biological and clinical mechanisms. Despite the cohort differences in tumor grade, bone metastases, and subtypes, the nomogram maintained robust performance, highlighting its resilience and generalizability.

The nomogram demonstrated a robust predictive performance, with C-index values ranging from 0.740 to 0.787 across cohorts and time-dependent AUCs consistently above 0.70. Calibration curves showed close agreement between predicted and observed survival probabilities, and DCA indicated clinically meaningful net benefits across a range of threshold probabilities. The external cohort, while partially overlapping temporally with the SEER data, was geographically and institutionally distinct, supporting external validation; however, a minor temporal bias should be considered. These results highlight the ability of the nomogram to stratify patients according to risk and provide individualized survival predictions, demonstrating a promising predictive performance.

Key prognostic variables have a well-established biological relevance. Advanced age is associated with comorbidities, immunosenescence, and impaired antitumor immunity, which contribute to poor outcomes (10, 11). Larger tumors and higher T stages increase the likelihood of vascular and lymphatic invasion, genetic instability, and resistance to therapy (12, 13). Multiple metastases, particularly brain involvement, indicate aggressive tumor biology, immune evasion, and metastatic adaptability, leading to shorter survival (13, 14). Higher histological grade reflects proliferative activity and genomic instability (15–17). ER and PR positivity indicates a more favorable prognosis due to endocrine therapy responsiveness (18), whereas HER2-positive tumors remain clinically challenging due to higher proliferation rates and partial resistance to targeted therapies (19, 20). Chemotherapy confers survival benefits through cytotoxic and immunogenic mechanisms, reinforcing its importance in treatment planning (21, 22).

The present study offers several advantages over existing BCLM prognostic models. Previous models were limited to conventional statistical approaches, lacked machine learning–assisted feature selection, and restricted external validation (9, 23, 24). Our nomogram incorporates a larger cohort, applies rigorous feature selection, and demonstrates reproducible performance across independent datasets. Compared with prior nomograms, the C-index improved by approximately 0.05, highlighting enhanced discriminative ability. These findings underscore the robustness and potential clinical utility of this model, although direct head-to-head comparisons with previous models should be conducted in future studies.

This study has some limitation. First, it was retrospective and based on SEER data, which may have introduced a selection bias due to limited treatment details and missing variables such as liver function, tumor markers, comorbidities, and lifestyle factors, potentially affecting the model accuracy. In addition, patients with incomplete clinical information or interrupted follow-ups were excluded during cohort selection, which may have resulted in a study population that was relatively healthier or had more complete data, potentially leading to an overestimation of the model performance. The external validation cohort included only 110 patients, which may have widened the CIs and limited generalizability. Moreover, the external cohort spans 2014–2024, partially overlapping with the SEER data (2010–2021) and introducing temporal bias rather than fully independent external validation. Despite these limitations, the model performance remained stable. Larger, multicenter studies using entirely non-overlapping cohorts, as well as additional validation in non-white and international populations, are warranted to further confirm the robustness of the nomogram and broaden its clinical applicability.

In conclusion, this study presents a rigorously developed and externally validated nomogram to predict survival in patients with BCLM. By integrating the clinicopathological variables identified through machine learning and Cox regression, the model enables individualized risk stratification and informs clinical decision-making. The nomogram demonstrates promising predictive performance and may have potential clinical utility, pending further validation. Future prospective multicenter studies are warranted to confirm and refine the applicability of these findings.

## Data Availability

The original contributions presented in the study are included in the article/supplementary material, further inquiries can be directed to the corresponding author/s.

## Glossary

AUC: area under the curve
BCLM: breast cancer liver metastasis
CI: confidence interval
C-index: concordance index
DCA: decision curve analysis
DT: decision tree
ER: estrogen receptor
HER2: human epidermal growth factor receptor 2
HR: hazard ratio
IHC: immunohistochemistry
IQR: interquartile range
ISH: *in situ* hybridization
LASSO: least absolute shrinkage and selection operator
OS: overall survival
PR: progesterone receptor
RF: random forest
ROC: receiver operating characteristic
SEER: Surveillance, Epidemiology, and End Results
TNM: tumor-node-metastasis
XGBoost: extreme gradient boosting
